# Supplementary material for: End Sequence Analysis Toolkit (ESAT) expands the extractable information from single-cell RNA-seq data
Source: Genome Res. 2016 Oct;26(10):1397–410. doi: 10.1101/gr.207902.116 (PMC5052061; doi:10.1101/gr.207902.116)
Supplement: Supplemental Material [file supp_gr.207902.116_Supp_Materials.tar.gz › ESAT-esat_v0.1/doc/html/classumms_1_1core_1_1model_1_1score_1_1_poisson_enrichment_score.html]

ESAT: umms.core.model.score.PoissonEnrichmentScore Class Reference


ESAT


- Main Page
- Related Pages
- Packages
- Classes
- Files

- Class List
- Class Index
- Class Hierarchy
- Class Members

All Classes Namespaces Files Functions Variables Enumerator Pages

- umms
- core
- model
- score
- PoissonEnrichmentScore

Classes |
Public Member Functions |
Static Public Attributes |
List of all members

umms.core.model.score.PoissonEnrichmentScore Class Reference

Inheritance diagram for umms.core.model.score.PoissonEnrichmentScore:

[legend]

Collaboration diagram for umms.core.model.score.PoissonEnrichmentScore:

[legend]

|  |  |
| --- | --- |
| Classes | |
| class | **Processor** |
|  | |

|  |  |
| --- | --- |
| Public Member Functions | |
|  | PoissonEnrichmentScore (AlignmentModel sample, AlignmentModel ctrl, Annotation a) |
|  | |
|  | PoissonEnrichmentScore (AlignmentModel sample, Annotation a) |
|  | |
| double | calculatePVal (double a, double b, double lambdaSample, double lambdaCtrl, double w) |
|  | |
| double | getAverageCoverage (AlignmentModel data, CoordinateSpace coordSpace) |
|  | |
| void | setPvalue (double scanPvalue) |
|  | |
| double | getPvalue () |
|  | |
| void | setGlobalLength (double d) |
|  | |
| void | setRegionLength (double regionLength) |
|  | |
| double | getGlobalLength () |
|  | |
| double | getRegionLength () |
|  | |
| double | getSampleLambda () |
|  | |
| double | getCtrlLambda () |
|  | |
| double | getLocalSampleLambda () |
|  | |
| double | getCtrlSampleLambda () |
|  | |
| void | setSampleCount (double d) |
|  | |
| void | setCtrlCount (double d) |
|  | |
| void | setSampleTotal (double d) |
|  | |
| void | setCtrlTotal (double d) |
|  | |
| void | setSampleRegionTotal (double d) |
|  | |
| void | setCtrlRegionTotal (double d) |
|  | |
| double | getSampleCount () |
|  | |
| double | getCtrlCount () |
|  | |
| double | getSampleTotal () |
|  | |
| double | getCtrlTotal () |
|  | |
| double | getSampleRegionTotal () |
|  | |
| double | getCtrlRegionTotal () |
|  | |
| Public Member Functions inherited from umms.core.model.score.CountScore | |
|  | CountScore (Annotation a) |
|  | |
|  | CountScore (CountScore other) |
|  | |
|  | CountScore (CountScore other, Annotation newAnnotation, double newCount) |
|  | |
|  | CountScore (AnnotationCollection<?extends Annotation > model, Annotation annotation) |
|  | |
|  | CountScore (AnnotationCollection<?extends Annotation > model, Annotation annotation, double regionTotal) |
|  | |
|  | CountScore (AnnotationCollection<?extends Annotation > model, Annotation annotation, boolean fullyContained) |
|  | |
|  | CountScore (AnnotationCollection<?extends Annotation > model, Annotation annotation, double regionTotal, boolean fullyContained) |
|  | |
|  | CountScore (Annotation annotation, double count, double regionTotal, double total) |
|  | |
| double | getCount () |
|  | |
| double | getScore () |
|  | |
| double | getTotal () |
|  | |
| double | getRegionTotal () |
|  | |
| double | getRPKM () |
|  | |
| double | getRPK () |
|  | |
| void | setCount (double count) |
|  | |
| void | setTotal (double total) |
|  | |
| void | setRegionTotal (double regionTotal) |
|  | |
| String | toString () |
|  | |
| boolean | equals (Object o) |
|  | |
| int | compareTo (CountScore o) |
|  | |
| int | hashCode () |
|  | |
| void | refreshPvalue () |
|  | |
| double | getPvalue () |
|  | |
| Public Member Functions inherited from umms.core.model.score.WindowScore.AbstractWindowScore | |
|  | AbstractWindowScore (Annotation t) |
|  | |
| Annotation | getAnnotation () |
|  | |

|  |  |
| --- | --- |
| Static Public Attributes | |
| static double | DEFAULT\_REGION\_TOTAL = -1.0 |
|  | |
| Static Public Attributes inherited from umms.core.model.score.CountScore | |
| static double | DEFAULT\_REGION\_TOTAL = -1.0 |
|  | |

|  |  |
| --- | --- |
| Additional Inherited Members | |
| Static Public Member Functions inherited from umms.core.model.score.CountScore | |
| static double | asRPKM (double count, double total, int windowSize) |
|  | |
| Protected Attributes inherited from umms.core.model.score.WindowScore.AbstractWindowScore | |
| Annotation | annotation |
|  | |

## Detailed Description

Author
:   shari This class represents Poisson enrichment score for ChIP/CLIP from Mikkelsen et al. 2010. Can take either sample and control models or just sample.

## Constructor & Destructor Documentation

|  |  |  |  |
| --- | --- | --- | --- |
| umms.core.model.score.PoissonEnrichmentScore.PoissonEnrichmentScore | ( | AlignmentModel | *sample*, |
|  |  | AlignmentModel | *ctrl*, |
|  |  | Annotation | *a* |
|  | ) |  |  |

Here is the call graph for this function:

|  |  |  |  |
| --- | --- | --- | --- |
| umms.core.model.score.PoissonEnrichmentScore.PoissonEnrichmentScore | ( | AlignmentModel | *sample*, |
|  |  | Annotation | *a* |
|  | ) |  |  |

## Member Function Documentation

|  |  |  |  |
| --- | --- | --- | --- |
| double umms.core.model.score.PoissonEnrichmentScore.calculatePVal | ( | double | *a*, |
|  |  | double | *b*, |
|  |  | double | *lambdaSample*, |
|  |  | double | *lambdaCtrl*, |
|  |  | double | *w* |
|  | ) |  |  |

Parameters
:   |  |  |
    | --- | --- |
    | a | Sample reads |
    | b | Control reads |
    | lambdaSample | Sample reads total / Genome size (ie expected reads per base) |
    | lambdaCtrl | Control reads total / Genome size (ie expected reads per base) |
    | w | window size |

Returns
:   Poisson pvalue

Here is the caller graph for this function:

|  |  |  |  |
| --- | --- | --- | --- |
| double umms.core.model.score.PoissonEnrichmentScore.getAverageCoverage | ( | AlignmentModel | *data*, |
|  |  | CoordinateSpace | *coordSpace* |
|  | ) |  |  |

Here is the call graph for this function:

|  |  |  |  |  |
| --- | --- | --- | --- | --- |
| double umms.core.model.score.PoissonEnrichmentScore.getCtrlCount | ( |  | ) |  |

Here is the caller graph for this function:

|  |  |  |  |  |
| --- | --- | --- | --- | --- |
| double umms.core.model.score.PoissonEnrichmentScore.getCtrlLambda | ( |  | ) |  |

Here is the call graph for this function:

Here is the caller graph for this function:

|  |  |  |  |  |
| --- | --- | --- | --- | --- |
| double umms.core.model.score.PoissonEnrichmentScore.getCtrlRegionTotal | ( |  | ) |  |

Here is the caller graph for this function:

|  |  |  |  |  |
| --- | --- | --- | --- | --- |
| double umms.core.model.score.PoissonEnrichmentScore.getCtrlSampleLambda | ( |  | ) |  |

Here is the call graph for this function:

|  |  |  |  |  |
| --- | --- | --- | --- | --- |
| double umms.core.model.score.PoissonEnrichmentScore.getCtrlTotal | ( |  | ) |  |

Here is the caller graph for this function:

|  |  |  |  |  |
| --- | --- | --- | --- | --- |
| double umms.core.model.score.PoissonEnrichmentScore.getGlobalLength | ( |  | ) |  |

Here is the caller graph for this function:

|  |  |  |  |  |
| --- | --- | --- | --- | --- |
| double umms.core.model.score.PoissonEnrichmentScore.getLocalSampleLambda | ( |  | ) |  |

Here is the call graph for this function:

|  |  |  |  |  |
| --- | --- | --- | --- | --- |
| double umms.core.model.score.PoissonEnrichmentScore.getPvalue | ( |  | ) |  |

Here is the caller graph for this function:

|  |  |  |  |  |
| --- | --- | --- | --- | --- |
| double umms.core.model.score.PoissonEnrichmentScore.getRegionLength | ( |  | ) |  |

Here is the caller graph for this function:

|  |  |  |  |  |
| --- | --- | --- | --- | --- |
| double umms.core.model.score.PoissonEnrichmentScore.getSampleCount | ( |  | ) |  |

Here is the call graph for this function:

Here is the caller graph for this function:

|  |  |  |  |  |
| --- | --- | --- | --- | --- |
| double umms.core.model.score.PoissonEnrichmentScore.getSampleLambda | ( |  | ) |  |

Here is the call graph for this function:

Here is the caller graph for this function:

|  |  |  |  |  |
| --- | --- | --- | --- | --- |
| double umms.core.model.score.PoissonEnrichmentScore.getSampleRegionTotal | ( |  | ) |  |

Here is the call graph for this function:

Here is the caller graph for this function:

|  |  |  |  |  |
| --- | --- | --- | --- | --- |
| double umms.core.model.score.PoissonEnrichmentScore.getSampleTotal | ( |  | ) |  |

Here is the call graph for this function:

Here is the caller graph for this function:

|  |  |  |  |  |  |
| --- | --- | --- | --- | --- | --- |
| void umms.core.model.score.PoissonEnrichmentScore.setCtrlCount | ( | double | *d* | ) |  |

Here is the caller graph for this function:

|  |  |  |  |  |  |
| --- | --- | --- | --- | --- | --- |
| void umms.core.model.score.PoissonEnrichmentScore.setCtrlRegionTotal | ( | double | *d* | ) |  |

|  |  |  |  |  |  |
| --- | --- | --- | --- | --- | --- |
| void umms.core.model.score.PoissonEnrichmentScore.setCtrlTotal | ( | double | *d* | ) |  |

Here is the caller graph for this function:

|  |  |  |  |  |  |
| --- | --- | --- | --- | --- | --- |
| void umms.core.model.score.PoissonEnrichmentScore.setGlobalLength | ( | double | *d* | ) |  |

Here is the caller graph for this function:

|  |  |  |  |  |  |
| --- | --- | --- | --- | --- | --- |
| void umms.core.model.score.PoissonEnrichmentScore.setPvalue | ( | double | *scanPvalue* | ) |  |

Here is the caller graph for this function:

|  |  |  |  |  |  |
| --- | --- | --- | --- | --- | --- |
| void umms.core.model.score.PoissonEnrichmentScore.setRegionLength | ( | double | *regionLength* | ) |  |

|  |  |  |  |  |  |
| --- | --- | --- | --- | --- | --- |
| void umms.core.model.score.PoissonEnrichmentScore.setSampleCount | ( | double | *d* | ) |  |

Here is the call graph for this function:

|  |  |  |  |  |  |
| --- | --- | --- | --- | --- | --- |
| void umms.core.model.score.PoissonEnrichmentScore.setSampleRegionTotal | ( | double | *d* | ) |  |

Here is the call graph for this function:

|  |  |  |  |  |  |
| --- | --- | --- | --- | --- | --- |
| void umms.core.model.score.PoissonEnrichmentScore.setSampleTotal | ( | double | *d* | ) |  |

Here is the call graph for this function:

## Member Data Documentation

double umms.core.model.score.PoissonEnrichmentScore.DEFAULT\_REGION\_TOTAL = -1.0

static

---

The documentation for this class was generated from the following file:

- java/umms/core/model/score/PoissonEnrichmentScore.java


---

Generated on Fri Aug 15 2014 15:11:13 for ESAT by  

 1.8.7
